# Antibody Responses to Bovine Alphaherpesvirus 1 (BoHV-1) in Passively Immunized Calves

**DOI:** 10.3390/v11010023

**Published:** 2019-01-02

**Authors:** Stefano Petrini, Carmen Iscaro, Cecilia Righi

**Affiliations:** National Reference Laboratory for Infectious Bovine Rhinotracheitis (IBR), Istituto Zooprofilattico Sperimentale Umbria-Marche “Togo Rosati”, 06126 Perugia, Italy; c.iscaro@izsum.it (C.I.); c.righi@izsum.it (C.R.)

**Keywords:** IBR, passive immunity, calves

## Abstract

To date, in countries where infectious bovine rhinotracheitis (IBR) is widespread, its control is associated with deleted *marker* vaccines. These products lack one or more genes responsible for the synthesis of glycoproteins or enzymes. In Europe, the most widely used *marker* vaccine is one in which glycoprotein E (gE−) is deleted, and it is marketed in a killed or modified-live form. Using this type of immunization, it is possible to differentiate vaccinated animals (gE−) from those infected or injected with non-deleted (gE+) products using diagnostic tests specific for gE. The disadvantage of using modified-live gE-products is that they may remain latent in immunized animals and be reactivated or excreted following an immunosuppressive stimulus. For this reason, in the last few years, a new *marker* vaccine became commercially available containing a double deletion related to genes coding for gE and the synthesis of the thymidine-kinase (tk) enzyme, the latter being associated with the reduction of the neurotropism, latency, and reactivation of the vaccine virus. Intramuscularly and intranasally administered *marker* products induce a humoral immune response; however, the mother-to-calf antibody kinetics after vaccination with *marker* vaccines is poorly understood. This review discusses several published articles on this topic.

## 1. Introduction

Bovine alphaherpesvirus 1 (BoHV-1), belonging to the genus *Varicellovirus* in the subfamily *Alphaherpesvirinae* under the family *Herpesviridae,* is an important pathogen of cattle [1,2,3,4].

**Figure 1:**
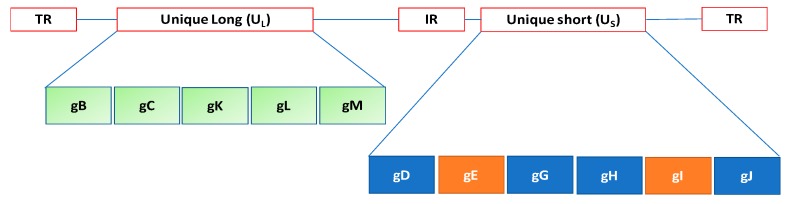
The genome structure of herpesviruses comprises two regions designated Unique Long (U_L_) and Unique Short (U_S_). Terminal repeat (T_R_) and Internal repeat (I_R_) sequences may bracket unique sequences of both L and S or only S. Each region encodes different envelope glycoproteins.

The virion structure of *Herpesviridae* is complex. The genome is composed of linear dsDNA ranging from 125 to 240 kb [5]. The genome structure of herpesviruses comprises two regions designated Unique Long (U_L_) and Unique Short (U_S_). Terminal repeat (T_R_) and Internal repeat (I_R_) sequences may bracket unique sequences (U_L_, U_S_) of both L and S or only S. Herpesvirus virions contain over 30 structural proteins, of which 6 are present in the nucleocapsid and 2 are DNA associated. In addition, about 11 glycoproteins are located in the envelope [6], from which most project as peplomers (Figure 1).

Moreover, based on genetic and antigenic analyses, there are three subtypes of BoHV-1: BoHV-1.1, BoHV-1.2a, and BoHV-1.2b [7]. These subtypes include BoHV-1.3a and BoHV-1.3b, which are now a separate species named BoHV-5 [3].

The virus is responsible for significant economic losses in the cattle industry worldwide, and several countries are working toward controlling or eradicating the infection [8,9].

The clinical symptoms of the disease are varied, and its severity depends on the virulence of the strain in circulation.

Subtypes BoHV-1.1 and BoHV-1.2a cause infectious bovine rhinotracheitis (IBR) and can be isolated from aborted fetuses [10]. Otherwise, BoHV-1.2b is responsible for infectious pustular vulvovaginitis (IPV) or infectious balanoposthitis (IBP), but it can also be associated with respiratory disease [11,12].

The virus can also result in a number of other clinical conditions, such as conjunctivitis, enteritis, and rarely encephalitis [13,14]. In addition, neonatal calves exposed to BoHV-1 were found to have a fatal multisystemic form involving the respiratory, gastrointestinal, nervous, and lymphatic systems [15].

BoHV-1 establishes latency in ganglia or tonsils, following primary infection in nasal cavities, or in the sacra ganglia, following genital infection [16,17]. BoHV-1 can be periodically reactivated, and the virus is shed and transmitted [18].

To date, in countries with a high prevalence of infection, IBR is controlled by the use of conventional modified-live (MLV) and killed vaccines (KV) as well as subunit vaccines. In several European countries the deleted *marker* vaccines are also used [8,19,20,21,22]. These products lack one or more viral genes responsible for the synthesis of enzymes or glycoproteins. In particular, the gE-deleted *marker* vaccines (killed or modified live) lack the gene responsible for the synthesis of glycoprotein E (gE) of BoHV-1. This glycoprotein forms a heterodimer with glycoprotein I (gI) and constitutes an Fc receptor, which has been implicated in the disruption of the host immune response. The gE-gI complex facilitates the basolateral spread of progeny viruses in polarized cells, suggesting its role in virion transport [23].

In recent years, a new doublegene-deleted IBR *marker* vaccine (modified-live) has become commercially available, and the viral genes coding for gE and thymidine-kinase enzyme (tk) have been modified. The tk gene was selected because it was reported to reduce viral neurotropism, therefore reducing the risk of latency and reactivation [8,24,25].

The use of gE-deleted *marker* vaccines makes it possible to serologically discriminate between vaccinated and infected animals, and they can be used to implement control schemes for BoHV-1 in European countries [8,26,27,28]. In addition, other types of *marker* vaccines are available, such as (1) modified-live gG/tk-; (2) killed; (3) gC-live; (4) gD-subunit; (5) gB-subunit; and (6) gD-replication-incompetent [29,30,31,32,33].

BoHV-1 may infect seronegative animals and remain within the population, creating so-called “seronegative latent carriers” (SNLCs) resulting from infected or passively immunized calves.

For the above-mentioned reasons, the present review on IBR vaccines focuses on passive immunity in calves from non-*marker* and *marker* vaccines.

## 2. Passive Immunity from Non-*Marker* Vaccines

The first commercial vaccine against IBR virus was developed many years ago [34,35], and subsequently, attenuated viruses with minimum licensing requirements were defined [36,37,38].

### 2.1. Colostrum

**Figure 2:**
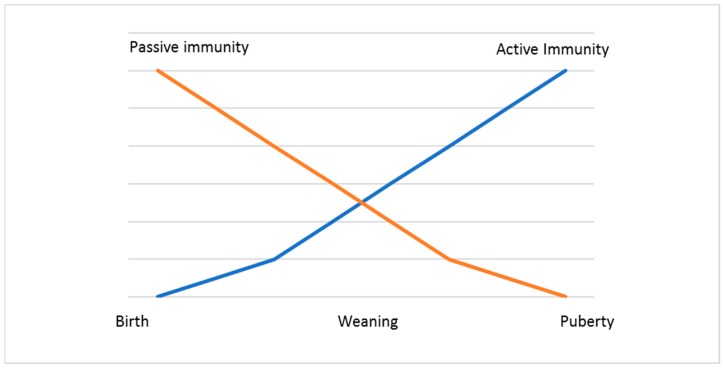
Development of the immune response in calves.

In cattle, the immunoglobulin G subtype 1 (IgG_1_) and not immunoglobulin A (IgA) is the predominant secretory immunoglobulin in colostrum and milk [39]. When calves are born, they are in an agammaglobulinemic status and thus are highly dependent on the efficient external uptake of maternal IgG from colostrum, in which these immunoglobulins are the prevalent proteins [40,41]. They have no antibodies in circulation or in their tissues. For this reason, the ingestion of colostrum to provide neonates with protection during the first weeks of their life is essential (Figure 2).

If the quality of the colostrum is not high in IgG, calves can develop a pathological condition called “failure of passive transfer” (FPT) and at an increased risk of mortality within the first 3 months of life [42,43]. In order to prevent FTP, calves should be given colostrum supplemented with IgG at a dose of 100–150 g per 1.5–2.0 L immediately after birth [44]. To measure IgG levels, different authors have applied the radial immunodiffusion technique to serum taken 2 days after birth [45,46]. Failed passive transfer of immunoglobulins is defined as a total of serum IgG concentration of less than 10 g/L at 2 days of age [46]. In addition, the apparent efficiency of adsorption (AEA) of IgG can be calculated [47,48].

### 2.2. Protection Provided by Colostrum

Antibodies in colostrum represent a critical component to protect calves from BoHV-1 because they produce immunoglobulin M (IgM) up to 4 days after birth and their functionality is not activated until 8 days of age. The levels of IgA, IgG_1_, and IgG_2_ do not reach detectable levels in calves that ingest colostrum until 16 to 32 days after birth. Their antibodies do not reach adult levels until approximately 4 months after birth and are mainly represented by IgG_2_ [49].

### 2.3. Passive Immunity

It has been demonstrated that passive immunity transferred from bovines immunized with intramuscular or intranasal non-*marker* vaccines protects calves from the respiratory form of IBR and decreases the severity of the pathological changes associated with the disease [50]. In addition, this immunity protects animals from re-infection with BoHV-1 [51] and significantly reduces virus shedding [52].

Colostrum antibodies prevent the systematic virus localization and, possibly, even viremia. In addition, they can significantly reduce the growth of the vaccine virus in the upper respiratory tract [15]. Specific colostral antibodies against IBR virus appear in the nasal secretion of calves as early as the first day after ingestion of colostrum. The colostral antibodies secreted on the respiratory tract mucosa, primarily pertaining to the IgG_1_ class, persist for 15 to 20 days after birth, whereas the serum antibodies may be detected until the calf is 4 to 6 months of age [50,53,54,55,56]. In addition, antibodies that neutralize BoHV-1 are transferred to calves from dams vaccinated with a non-*marker* vaccine [15].

To increase the concentration of maternal antibodies in colostrum, several authors have suggested pre-partum vaccination [57]. Indeed, animals with higher serum antibody titers also partitioned higher concentrations of such antibodies to the mammary glands, whereas there was variation in the concentration of specific colostral antibodies [58,59,60]. Furthermore, the use of multivalent KV in pregnant cattle induced an increase in the maternal antibodies 5 weeks after the first injection and 3 weeks after the booster vaccine, antibodies that can be transferred via colostrum to the calves. In fact, using this practice, on parturition day, significantly higher titers of antibodies can be transferred from vaccinated dams to calves [57,58]. However, different studies have reported the duration of detection of maternal colostral antibodies against BoHV-1 as the mean time to reach seronegativity for BoHV-1. One study on a group of calves that had received maternal colostrum from dams immunized with MLV vaccine (Titanium MLV Cattle Vaccines, Agri Laboratories, St. Joseph, MO, USA), estimated this mean time as 65.1 ± 37.8 days [55]. In another study where a MLV vaccine (Titanium 5 L5, Agri Laboratories, St. Joseph, MO, USA) was used, the mean duration of colostrum-derived antibodies in calves was estimated to be 122 ± 46.6 days [61]. However, other studies involving the use of a killed vaccine, reported the mean BoHV-1 antibody level to be significantly higher at 8 months of age in calves fed on maternal-colostrum, although the standard deviation for time to seronegativity ranged from 1.5 months (45 days) to 1.7 months (53 days) [45].

The longevity of passively acquired immunity in calves that received maternal colostrum at birth is highly variable [55,61,62,63]. The duration of colostrum-derived immunity and the presence of calves seronegative to BoHV-1 could result in poor animal immunity and increase the risk of virus introduction into a herd. The variability in the duration of colostrum-derived immunity against BoHV-1 is related to different factors, including differences in the rate of decay of colostrum-derived antibodies [55,61,62,63,64], which is usually influenced by active viral infection or vaccination. Different studies [63] have reported that the half-life of maternally transferred antibodies to IBR after vaccination with a modified-live vaccine in dams was 21.2 days. In contrast, the half-life of maternally transferred antibodies to IBR calves vaccinated at branding and weaning (about 95 days of age) with KV was found to be 31.8 days. In addition, other studies reported that the BoHV-1 colostral antibody half-life in calves was 19, 21, and 23 days [63,65,66]. Otherwise, the length of time to become seronegative for calves not vaccinated with maternal antibodies was estimated as 122.9 days, whereas for vaccinated calves, it was 169 days [65].

Maternal-derived immunity may block the serum antibody response. Otherwise, vaccinated seronegative calves respond with an active humoral immune response to BoHV-1. In addition, calves with low antibody titers to BoHV-1 may not respond to MLV vaccines or to KV [7,55]. When an MLV BoHV-1 vaccine was used, calves with maternal antibodies did not seroconvert after the initial vaccination but were primed for a secondary response after the subsequent vaccination. This pattern could be due to virus-specific T-cell responses, such as when calves with BoHV-1 antibodies developed T-cell responses even in the absence of increased antibody levels to BoHV-1 [67]. In general, there is evidence that maternal immunity blocked antibody production even when two doses were given to the calves with MLV (Triangle 4^®^), especially in animals with high maternally derived antibody titers [63].

In one study on passively immunized calves, no antibody response to BoHV-1 inoculation was detected, except for in a single animal [68]. In particular, IgG_2_ were detected 14 days post-infection. These immunoglobulins were found to be four two-fold dilutions lower than the IgG_1_ response at 35 days post-infection [68,69,70]. The suppression of the antibody response by maternal antibodies is caused by infection with virulent BoHV-1 [71].

### 2.4. Vaccines

As shown by several authors, the best time to vaccinate calves without interfering with maternal immunity is the period between 16 and 28 days after birth, when the maternal antibodies decay [63]. In addition, good vaccination depends on the level of maternal antibodies and the vaccine antigen, which presents a major challenge for vaccine development. Three or four months of age can be a good time to administer an MLV.

However, parenteral vaccination with a quadrivalent vaccine at 10 days of age followed by a booster at 6 months did not give rise to an antibody response to BoHV-1 [66]. Otherwise, parenteral vaccination with either an MLV or a KV at 7 weeks of age in the presence of maternal antibodies resulted in a response in the MLV group with no increase in antibody titers for either vaccine. Booster with either vaccine 4.5 months later resulted in a response in antibody titers, which indicated the importance of timing [71].

A vaccination strategy that avoids interference with maternal antibodies consists of vaccinating young calves intranasally (IN) [72,73,74]. Immunity produced after IN vaccination can protect them for several months. Moreover, this type of immunization can induce interferon within 40 h after administration, thus providing an antiviral action and stimulating the immune system.

IN vaccination against BoHV-1 has the following characteristics: (i) very low interference with maternal immunity; (ii) high production of interferon in mucosal sites and serum, and the interferon has an antiviral effect and leads to development of the neonatal immune response; (iii) no development of immunosuppression as for IM vaccines; and (iv) can result in latency [72,75,76].

The risk of IN vaccination with MLV in young calves is the spread of vaccine virus in the environment. In this case, the vaccine virus can be isolated from unvaccinated calves, living with vaccinated animals. Also, the vaccine virus does not revert to virulence during passage in calves, and it is unlikely that the vaccine strain gives rise to latency [26].

In general, vaccination schemes require that calves must be vaccinated according to protocols during the first 2 months of age. For calves, vaccination frequency is critical to ensure a good immune response. Indeed, too frequent vaccinations in young calves can lead to antigen-specific tolerance, represented by the suppression of T cells and deletion of T and B cells or cause autoimmunity. In particular, antibodies against different proteins of IBR cross-react with a surface protein of immune cells [74].

### 2.5. Seronegative Latent Carriers (SNLCs)

Latently infected animals are usually evidenced by the detection of BoHV-1-specific antibodies in their serum samples when the virus is reactivated. In this case, the humoral response observed after the reactivation of the latent virus is similar to the one that occurs after the primary infection. In particular, it is possible to observe the production of the neutralizing antibodies about 10–14 days after viral reactivation [77,78].

However, it has been postulated that some infected animals contain a low quantity of antibodies. After infection, if no antibody response is produced, an SNLC might be generated [70]. However, maternal antibodies can interfere with a humoral immune response following infection or vaccination. Different authors have conducted several studies to demonstrate this hypothesis. In particular, in a study on *marker* vaccines, the effects in neonatal calves of an injected modified-live attenuated gE-negative *marker* vaccine were investigated. The results indicated that the immunized gE-negative calves produced an antibody response to gE after infection with virulent BoHV-1 and that the virus was reactivated [79].

In calves, as demonstrated in another study, the presence of maternal antibodies did not prevent the viral replication or latency of the virulent BoHV-1 used. In addition, no increase in antibodies was found following infection, and the results suggested the origin of SNLCs [70]. Moreover, passive immunity did not prevent virus excretion and establishment of latent infection [75].

Furthermore, it was determined whether BoHV-1 SNLCs can be experimentally obtained after infection of immunized calves with an MLV temperature-sensitive (*ts*) BoHV-1 vaccine. The *ts* vaccine produced acute and latent infections in vaccinated calves in both the presence and absence of passive immunity. The results indicated that SNLCs can be generated by an MLV vaccine under passive immunity [75,79,80,81].

Moreover, several authors have provided evidence for the absence in some animals of detectable seroconversion after immunization with MLV BoHV-1 strains, particularly with the *ts* vaccine. The failure of that particular calves to evidence serological response to IBR is atypical, and it suggests that the animals were immunodeficient. However, in this case, and before the vaccination, the latent state has never been studied [82,83].

Differently, in a further study calves vaccinated with a MLV *ts* mutant after challenge were inoculated intravenously with dexamethasone for 5 days. The results showed that some calves reactivated the field virus while others the vaccine virus [84].

In another study, involving the use of different vaccines including MLV *ts* mutant, after vaccination and challenge, dexamethasone was administered intramuscularly and the calves excreted only challenge virus [85].

An interesting result was observed regarding virus excretion. Indeed, the virus was detected for long periods of time in the presence of maternal antibodies. Regarding the viral excretion of the live attenuated gE negative vaccine, it was shown that it was reduced in calves with maternal immunity [81,86].

In addition, SNLCs could remain seronegative for long periods of time of up to 3–5 months based on the ELISA used. From 6 to 9 months, SNLCs could increase their immune response following the virus reactivation [87].

To identify SNLCs, in living animals, dexamethasone treatment can be used, while in dead calves, PCR or real-time PCR on trigeminal ganglion can be used to detect the gene coding the glycoprotein B (gB) of BoHV-1 [80].

SNLCs are economically important in BoHV-1 regions where control programs are employed. In addition, failing to detect these animals could represent a potential risk for artificial insemination centers, genetic selection stations, and BoHV-1-free herds or regions.

## 3. Passive Immunity from *Marker* Vaccines

The modified-live gE *marker* vaccine has an important role in evading the humoral immune response, including maternal antibodies [76].

To date, the use of conventional IBR vaccines interferes with the European programs designed to eradicate the disease; otherwise, in North America, only MLV and KV vaccines are used [88]. However, these products have several disadvantages regarding their safety and/or efficacy, which make them unsuitable for vaccination of some targets such as pregnant cows [89]. For these reasons, new strategies for vaccine development against BoHV-1 have been focused on the design of *marker* vaccines.

Marker vaccines make it possible to differentiate serologically between BoHV-1-vaccinated and BoHV-1-infected cattle. Seronegative cattle or animals vaccinated with a gE-negative vaccine developed specific antibodies against gE within 10–14 days after BoHV-1 infection [90,91]. *Marker* vaccines are used in association with specific ELISA tests that are capable of detecting the gE missing in the vaccine strain [92,93,94,95]. These products have been available since the 1990s [96,97,98,99]. Different types of *marker* vaccines are available, such as gE-live, gE-killed, gG-killed, gC-live, gD-subunit, gB-subunit, and gD-replication-incompetent vaccines [30,32,98]. Some of these have been authorized by the European Medicine Agency (EMA; Table 1). In particular, vaccines with a deleted gene coding for non-essential glycoprotein E (gE) or the synthesis of thymidine-kinase enzyme (tk) are commercially available [8,79]. gE, a non-essential glycoprotein for virus replication in vitro, is a membrane protein conserved among the alphaherpesviruses, which suggests its importance in vivo [99]. In addition, gE might play an important role in evading the humoral immune response, including maternal antibodies.

In particular, gE forms a heterodimer with gI, and this complex constitutes an Fc receptor, which is implicated in the disruption of the host immune defense. Moreover, the gE-gI complex facilitates the basolateral spread of progeny viruses in polarized cells, suggesting its possible role in virion transport [23].

A new commercially available deleted vaccine contains two deletions constructed with a virus isolated from an outbreak of IBR. The virus was modified to alter the genes coding for gE and the thymidine-kinase (tk) enzyme. The tk gene was selected because is associated with viral neurotropism and latency. Reactivation can occur in infected animals, and therefore, an additional deletion comprising this gene would improve protection against the viral strain [100,101]. These deletions are known to reduce the virulence of IBR. The beneficial effects of this vaccine are the stimulation of active immunity against BoHV-1 in cattle from 3 months of age and the reduction in clinical signs of IBR and virus shedding. The onset of immunity is 21 days after completion of the basic vaccination scheme, and the duration of immunity is 6 months after completion of the basic vaccination scheme [102]. In addition, gE- and tk/gE-deleted vaccines can be used in IBR eradication programs [8,96].

### 3.1. Antibody Response

When gE-negative *marker* vaccines are injected twice into seronegative dams, they induce maternal antibodies to BoHV-1 but no antibodies directed against gE. The possible presence of maternal antibodies to gE may be responsible for the production of an antibody response to the infection, inducing gE-negative latently-infected animals. In calves with maternal antibodies and subjected to infection, there was no immune response to BoHV-1 [79], while other authors did not demonstrate an increase in the antibody level after the BoHV-1 infection [70]. In addition, different authors have shown that passively immunized gE-negative young calves can develop an active and lasting antibody response to gE after infection with BoHV-1 [79,103]. Afterwards, the animals seroconvert to gE because the virus replicates in the nasal mucosa, and high levels of passively acquired antibodies do not prevent virus replication and establishment of latency [76,79,102]. However, different authors have reported that two calves receiving colostrum seronegative for gE did not develop gE antibodies after BoHV-1 infection [104]. The authors hypothesized that the low replication rate observed in these calves after infection did not interfere with the establishment of a latent state without a gE antibody response; further experiments were carried out, indicating that most likely the calves had not been lately infected. The calves seroconverted to gE within 2 to 5 weeks after they were infected with BoHV-1, and the antibodies to gE could be detected up to 6 months after experimental infection [26,52,96,103]. These results show that the duration of gE could depend on the use of a lower infectious dose used and on higher passive antibodies levels at the time of infection. Protection can be induced at as early as 7 days after intramuscular administration, and the vaccine can be administered early in an outbreak [52]. Inactivated vaccines induce a strong serum neutralization response, while a live attenuated gE-negative vaccine was demonstrated to induce the best protection, as evidenced by the absence of clinical signs [105]. Intranasal vaccination with a modified-live gE *marker* vaccine was shown to protect in the presence of maternally derived antibodies [52]. In contrast to live *marker* vaccines that induce short-term transient pyrexia and nasal discharge, the gE marker vaccine has been shown to be safe for use in breeding cattle [52,97].

### 3.2. Clinical Form

Partial protection against the clinical form of BoHV-1 was demonstrated [81]. The presence of maternal antibodies did not prevent virus shedding and the establishment of a latent state after BoHV-1 infection [68,71,103,105].

### 3.3. Latency

The establishment of BoHV-1 latency is associated with gE seroconversion after infection in the presence of high levels of passive immunity lacking gE antibodies [79]. During the primary or initial infection, BoHV-1 comes into contact with the receptors of local sensory nerves. BoHV-1 attaches to and penetrates the nerve cell via olfactory receptors located in the nasal mucosa. For BoHV-1, the infection is predominantly in the trigeminal nerve. After entering the nerve, the virus is transported by retrograde transport along the microtubules of the axon to the trigeminal ganglia and the tonsils 24–72 h post-infection [16,18].

Different authors have demonstrated that after dexamethasone (DMS) treatment, infectious BoHV-1 was isolated 5 months after infection, and some animals did not develop gE antibodies [103]. The authors hypothesized that the low replication level evidenced in these animals after infection did not interfere with the latent state without a gE antibody response. The presence of high levels of maternal antibodies without gE antibodies does not prevent latency after infection. Moreover, latency is associated with a serological response to gE.

BoHV-1 gE-negative vaccine strains can establish latency in passively immunized calves after a single intranasal inoculation [53,78,89,96,106,107]. Other studies have reported contrasting results [108,109,110].

### 3.4. Excretion

Calves vaccinated with an inactivated *marker* vaccine excreted less challenge virus [105]. Moreover, a modified-live *marker* vaccine (Bovilis IBR marker live used by intramuscular route) did not lead to shedding of the vaccine virus in nasal secretions, and no vaccinated animals had detectable viremia, whereas the virus was excreted for 1–8 days after intranasal administration [52,111]. The presence of maternal antibodies in calves did not prevent viral replication after infection and did not decrease the duration of shedding [112]. In addition, after infection, long periods of excretion were observed with BoHV-1, Suid aphaherpesvirus 1 (SuHV-1), or Human alphaherpesvirus 1 (HSV-1) in passively immunized mice. Several studies indicated that gE and its complex with glycoprotein I (gI) play an important role in humoral immune evasion [112,113]. Currently, the precise function of BoHV-1 gE is not clear. For SuHV-1, gE plays a role in virulence because gE deletion results in it being avirulent in calves [76,96,97,114]. BoHV-1 gE could be involved in an immune evasion mechanism. In particular, as reported for Human alphaherpesvirus 1 (HSV) and Human alphaherpesvirus 3 (VZV), the Fc receptor of the gE-gI complex can bind the Fc domain of IgG and consequently inhibit in vitro the Fc-mediated immune functions [115,116]. In the presence of specific antibodies, this Fc receptor from the gE-gI complex induces a bipolar bridge bond. IgG Fc binds the HSV-1 gE-gI complex in a process that is currently unknown [117]. The complex could remain at the cell surface or be endocytosed with IgG. The gE-gI complex can mediate the clearance of infected cell surfaces of anti-viral host IgG and viral antigens to evade IgG-mediated responses, representing a general mechanism for viral Fc receptors in immune evasion and viral pathogenesis. Even though it is generally accepted that all herpesviruses have highly immune-evasive properties, the Fc receptor activity has not been demonstrated for BoHV-1 [117,118]. In addition, different authors have suggested that the natural BoHV-1 infection of calves possessing maternally derived antibodies could represent a good model for the investigation of the immune evasive character of alphaherpesviruses. Moreover, animals passively immunized and infected with BoHV-1 reactivated and re-excreted the same virus after treatment with dexamethasone [112].

### 3.5. Disadvantages

The disadvantages of gene-deleted vaccines include under- or over-attenuation, depending on the deleted gene [107].

## 4. Conclusions

In this review, we summarized several reports on passive immunity from non-*marker* and *marker* vaccines against BoHV-1.

The colostrum and the maternal-derived antibody responses have been discussed for BoHV-1. Maternal immunization appears to be pivotal in preventing the clinical signs of BoHV-1 infection in young calves. However, to date, no vaccine is able to prevent the infection and the establishment of latency by challenge and field strains.

Many vaccines are available to control BoHV-1 infection in cattle. It is difficult to differentiate the animals vaccinated with conventional vaccines and animals with infection. So, the gE-deleted vaccines may play an important role in IBR eradication programs because this type of vaccination makes it possible to discriminate between vaccinated and infected animals. In addition, a commercially available gE-deleted vaccine containing two deletions (gE-,tk-) and constructed with a virus isolated from an outbreak of IBR, has been reported. Moreover, in the context of IBR eradication programs in European countries, BoHV-1 latently infected animals and SNLCs are important. In particular, more data of SNLCs need to be collected and studied. To date, little information is available about the immune response in calves with maternal-derived antibody. For these reasons, the SNLC animals represent a danger for BoHV-1 free herds, selection stations and artificial insemination centers.

Finally, further studies on maternal immunization programs are needed to evaluate new experimental vaccines.

## Figures and Tables

**Table 1:** Infectious bovine rhinotracheitis (IBR) *marker* vaccines available in European countries.

Name of Vaccine (Company)	Active Substance	Vaccine Strain	Dose	Route	Marketing Authorization Numbers
Hiprabovis IBR Marker Live (Hipra)	Live gE-, tk-, double-gene deleted BoHV-1 virus	Ceddel 10^6.3^–10^7.30^ CCID_50_^a^	Single 2 mL	i.m.	EMEA/V/C/000158
Cattlemarker IBR Inactivated (Zoetis)	gE-, inactivated virus	Difivac gE-, ≥5.5 log_2_^b^	Single 2 mL	s.c.	EMEA/V/A/115
Bayovac IBR Marker Vivum (Bayer)	gE -, modified live (attenuated) virus	Divifac 10^5^ TCID_50_ (min)–10^7^ TCID_50_ (max) ^c^	Single 2 mL	i.n., i.m.	EMEA/V/A/023b/001
Bovalto Ibraxion Inactivated IBR virus (Merial)	gE-, inactivated IBR virus	0.75 VN.U ^d^	Single 2 mL	s.c.	EMEA/V/C/000051

^a^ CCID_50_, cell culture infectious dose 50% endpoint; ^b^ log_2_, logarithm in base 2; ^c^ TCID_50_, tissue culture infectious dose 50% endpoint; ^d^ VN.U., virus neutralizing antibody titer after vaccination in guinea pigs; i.m., intramuscular route; s.c., subcutaneous route; i.n., intranasal route.
